# Habitat degradation relates to reduced immune function in nestlings, but not adults, of a tropical forest bird

**DOI:** 10.1007/s00114-025-02046-3

**Published:** 2025-11-26

**Authors:** Tamara Isabelle Sorg, Arne Hegemann, Laurence Cousseau, Gladys Nyakeru Kung’u, Janne Heiskanen, Petri Pellikka, Mwangi Githiru, Luc Lens, Beate Apfelbeck

**Affiliations:** 1 Department of Environment and Biodiversity, University of Salzburg, Salzburg, 5020 Austria; 2 Behavioral Ecology and Sociobiology Unit, German Primate Center, Göttingen, 37077 Germany; 3 Department of Sociobiology/Anthropology, Johann-Friedrich-Blumenbach Institute of Zoology and Anthropology, Georg-August-University Göttingen, 37077 Göttingen, Germany; 4 Department of Biology, Lund University, Lund, 223 62 Sweden; 5 Centre for Research on Ecology, Cognition and Behaviour of Birds, Ghent University, Ghent, 9000 Belgium; 6 Department of Geosciences and Geography, University of Helsinki, 00014 Helsinki, Finland; 7 Wildlife Works, Voi, P.O. Box 310-80300, Kenya; 8 Zoology Department, National Museums of Kenya, Nairobi, P.O. Box 40658–00100, Kenya; 9 Finnish Meteorological Institute, Helsinki, P.O. Box 503, 00101 Finland; 10 Finnish Southern Africa Cooperation Institute, 10 Schwabe Street, Windhoek, Namibia; 11 State Key Laboratory for Information Engineering in Surveying, Mapping and Remote Sensing, Wuhan University, Wuhan, 430079 China; 12 Institute of International Animal Health/One Health, Friedrich- Loeffler-Institute, Federal Research Institute for Animal Health, Greifswald - Insel Riems, 17493 Germany

**Keywords:** Bacteria killing capacity, Forest fragmentation, Forest degradation, Habitat change, Residual body mass, Return latency

## Abstract

Human-induced habitat loss, fragmentation and degradation are among the leading causes of species decline worldwide. This is particularly true in tropical forests, where unique, often highly specialized fauna is under threat. By altering resource availability, anthropogenic habitat change can impose resource constraints on animals and may influence their allocation of resources to competing life history traits. In this study, we investigated whether nestlings and adults differently invest in self-maintenance depending on habitat degradation in the placid greenbul (*Phyllastrephus cabanisi placidus*), a cooperative breeder native to the cloud forests of Eastern Africa. We quantified investment in self-maintenance by measuring innate immune function using bacteria killing assays (BKAs) in adult breeders and their nestlings along a gradient of fragmented and degraded forests of the Kenyan Taita Hills. While innate immune function is an important defence against pathogens, resources needed to maintain it may come at a cost to other processes such as nestling development. We show that while forest degradation did not affect adult innate immune function, nestlings bear the cost of growing up in degraded habitats, as their ability to clear bacteria from blood plasma was lower in areas with degraded vegetation. These findings highlight the importance of studying the effects of habitat fragmentation and degradation in the tropics, where most of the global biodiversity occurs, and where long-lived species may respond differently from short-lived temperate ones, for example by prioritising self-preservation over reproduction.

**Supplementary Information:**

The online version contains supplementary material available at 10.1007/s00114-025-02046-3.

## Introduction

Human activities are profoundly altering ecosystems and are a driving factor in the ongoing global loss of biodiversity (Newbold et al. 2015; Tilman et al. 2017). Tropical rainforests are suffering from unprecedented agricultural expansion and land-use intensification, leading to their degradation and fragmentation (Laurance et al. 2011; Martínez-Ramos et al. 2016). Such changes can affect vegetation composition, alter invertebrate communities and lead to altered availability and quality of trophic resources (Morante-Filho et al. 2016; Vergara et al. 2021; Cudney-Valenzuela et al. 2023). In addition, fragmentation can affect the prevalence of parasites, pathogens, and their vectors (Pérez-Rodríguez et al. 2018; Tchoumbou et al. 2020). As a result, many bird species may experience changes in food availability and pathogen exposure in fragmented habitats (Laurance et al. 2013; Pérez-Rodríguez et al. 2018; Dekeukeleire et al. 2019), with potential implications for their condition and health (Mazerolle and Hobson 2002). For example, logging and fragmentation of forests have been found to affect the production of glucocorticoid hormones and immunological traits in various bird and mammal species (Messina et al. 2018).

Reductions in resource availability caused by habitat fragmentation and subsequent degradation can particularly affect animals during reproduction, when resources must be allocated between investment in reproduction and self-maintenance (Drent and Daan 1980; Linden and Møller 1989; Stearns 1992). An important physiological component of self-maintenance is the immune system, which protects individuals from pathogens and increases survival by reducing disease-related mortality (Tieleman et al. 2005). However, it is also costly to maintain (Klasing 2004) and may be compromised when an animal is facing adverse conditions which can leave the animal vulnerable to pathogens (Strandin et al. 2018). Previous studies in several bird species have found a negative effect of increased reproductive effort on immune response and long-term fitness (Nordling et al. 1998; Ardia 2005; Hanssen et al. 2005; Knowles et al. 2009), demonstrating that reproductive effort is costly for self-maintenance. For example, experimentally increasing reproductive investment in a species with a fast pace of life led to a reduced immune response in breeders while maintaining offspring quality (Ardia 2005). Thus, when breeding conditions are poor, for example following habitat fragmentation and degradation, animals may need to increase their reproductive investment to successfully reproduce, and this may come at the expense of self-maintenance which can manifest in the form of a lowered immune defence.

Alternatively, the costs of breeding in low quality habitat may primarily affect nestlings, as parents may reduce reproductive investment to avoid compromising their own health (Gorman and Nager 2004). In addition, nestlings may be particularly vulnerable to changes in resource availability caused by habitat fragmentation and degradation because their immune systems are still developing (Mauck et al. 2005; Palacios et al. 2009; Aastrup and Hegemann 2021). Changes in diet composition, and therefore diet quality, are known to influence the development of both constitutive immune function (Hegemann et al. 2013) and induced immune responses (Lochmiller et al. 1993; Birkhead et al. 1999). Furthermore, changes in land cover have been negatively associated with constitutive innate immune function in nestlings of several grassland bird species (Merrill et al. 2019). However, it is currently unknown whether forest fragmentation and degradation differentially affect immune function of adults and nestlings in long-lived tropical forest species. Tropical birds are expected to prioritise self-maintenance over reproduction due to their slow pace of life (Wiersma et al. 2007), which is also manifested in higher levels of constitutive immune function of tropical compared to temperate birds (Tieleman et al. 2005).

In addition to environmental factors, individual quality may influence the trade-off between reproduction and self-maintenance and thus parental decision making. High quality individuals may cope better with environmental challenges and thus may be able to invest both in reproduction and maintenance of a high body condition, including strong immune defences (Ardia 2005; Roast et al. 2020). Especially during acutely stressful situations, individuals in poor condition may delay nestling provisioning or in species where nest desertion is naturally common even desert their brood (Ledwoń et al. 2019). Parental decisions in favour of self-maintenance may be especially common in long-lived species. As demonstrated in some long-lived seabird species and tropical house wrens (*Troglodytes musculus*), experimentally handicapped parents decreased their parental effort to maintain their own body condition (i.e., body weight or innate immune function) (Sæther et al. 1993; Tieleman et al. 2008).

The placid greenbul (*Phyllastrephus cabanisi placidus*) is an ideal taxon to test whether habitat fragmentation and degradation relate to investment into innate immune function in adults and nestlings of tropical birds. The placid greenbul is a tropical, long-lived, facultative cooperative breeder native to the cloud forests of East Africa, where one of its native habitats, the Kenyan Taita Hills, has suffered severely from fragmentation and degradation (Pellikka et al. 2009). In this study, we quantified innate immune function in both adults and nestlings along a gradient of fragmented and degraded native forests. Innate immune function is an important component of individual body condition and is the first line of defence against pathogens, consisting of proteins and lysozymes that are bactericidal and play an important role in limiting infection and thus increasing survival (Selsted and Martinez 1978; Esser 1994). Here, we used bacteria killing assays (BKAs), which quantify an individual’s ability to eliminate a pathogen (Matson et al. 2006; French and Neuman-Lee 2012) as an estimate of immune function strength. BKAs do not target specific proteins of immune function, but rather test an overall response of innate immune function against bacteria, and thus provide an integrative measure of innate immune function more than other more specific assays. Habitat fragmentation and degradation alter resource availability and hence presumed reproductive workload, and we hypothesize that placid greenbuls breeding in more degraded territories have to trade off self-maintenance against reproduction. Accordingly, we have previously found that placid greenbuls breeding in low quality forest areas have larger home ranges and higher corticosterone levels than those breeding in high quality forest areas (Apfelbeck et al. 2024; Kung’u et al. 2025), suggesting increased reproductive workload in low quality habitat. Because placid greenbuls are long-lived and produce small clutches, indicative of a slow pace of life typical of tropical species, we expect them to prioritise self-maintenance over reproduction. In line with this hypothesis, our analysis of a long-term dataset revealed that the body condition of nestlings, as measured by weight and wing length, is reduced in small forest fragments (Kung’u et al. under review). Thus, we expect that any negative effects of habitat fragmentation and degradation on innate immune function (as quantified by bacterial killing capacity) would be primarily manifested in nestlings, but not in adults. We test this by comparing (a) bacteria killing capacity (BKC) between adults and nestlings along a gradient of habitat quality and (b) by testing the behavioural response (resuming of nestling provisioning) of adults with varying body condition (i.e., immune function, residual body mass) to the stressor of capture and handling. We predict (i) no difference in adult BKC between territories that differ in degree of degradation. (ii) For nestlings, whose development depends on the food and resources provided, we expect that nestlings reared in degraded territories have weaker innate immune function and therefore lower BKC than nestlings reared in more intact habitat. (iii) Because individuals in poor condition (i.e., low BKC) are expected to invest more in self-maintenance than in reproduction, we expect a stronger behavioural stress response, i.e., they need longer to return to the nest to care for the nestlings after capture and handling, in individuals with low BKC compared to individuals with high BKC. We also compare residual body mass as an additional measure of body condition in birds caught in forest areas of varying quality assuming that residual body mass indicates overall nutrient stores (Labocha and Hayes 2012). In some studies habitat degradation was associated with lower residual body mass (Jones et al. 2022). However, residual body mass may also increase during food insecurity (Bateson et al. 2021) and reveal complex trade-offs between insurance against starvation risk and investment into immune function or reproduction (Lynn et al. 2010; Cornelius et al. 2017).

## Methods

### Study area

The study was carried out on the Dabida Massif in the Taita Hills (highest elevation 2208 m.a.s.l., elevational range of studied areas ~ 1550–1950 m.a.s.l.), located on the semi-arid Tsavo Plains in south-eastern Kenya (3°25’S, 38°20’E). Due to agricultural expansion, the indigenous cloud forests are highly fragmented. Approximately 50% of the indigenous forest was lost in the second half of the last century and only 13 cloud forest fragments remain (Chege and Bytebier 2005; Pellikka et al. 2009; Aerts et al. 2011). These vary greatly in size and quality. Larger and protected fragments are characterized by taller trees, higher tree species diversity (Schäfer et al. 2016), and better developed canopy cover than small, unprotected fragments with clear evidence that human disturbances further degrade the structural integrity of the vegetation (Kung’u et al. 2023). Furthermore, degraded vegetation structure is associated with reduced arthropod abundance (Kung’u et al. 2023). In this study, data were collected in two large fragments (Ngangao ~ 120–130 ha; Chawia ~ 86 ha), a severely degraded mixed native-exotic fragment (Vuria ~ 100 ha) and several smaller fragments (Fururu ~ 8 ha, Msidunyi ~ 21 ha, Ndiwenyi ~ 3 ha, Susu ~ 15 ha, and Iyale ~ 3 ha).

### Study species

The placid greenbul (*Phyllastrephus cabanisi placidus*) is a relatively long-lived passerine (Cousseau et al. 2020) native to the moist forests of East Africa. Sexes resemble each other, but males (mean ± sd weight 31,7 ± 2,0 g, mean ± sd tarsus length 27,5 ± 0,7 mm) are larger than females (mean ± sd weight 28,7 ± 1,8 g, mean ± sd tarsus length 26,6 ± 0,6 mm). It is insectivorous and gleans invertebrates from the bark and leaves of shrubs and climbers (Fishpool and Tobias 2020). It is a forest specialist and negatively affected by forest fragmentation (Lens et al. 2002). Breeding adults have larger home ranges and higher corticosterone levels in fragmented and degraded forest (Apfelbeck et al. 2024; Kung’u et al. 2025), and nestlings have a lower scaled mass index in such habitats (Kung’u et al. under review). Their breeding season extends from November to March coinciding with the rainy season in November and December. Placid greenbuls are sedentary facultative cooperative breeders and breeding pairs typically maintain their territories for several consecutive breeding seasons (Cousseau et al. 2020). Groups are formed mainly through delayed dispersal of offspring and vary in size from two (i.e., breeding pair only) to seven individuals (i.e., breeding pair plus five subordinates) (Cousseau et al. 2020, 2022). Subordinates typically assist the breeding pair by feeding nestlings and fending off predators, thereby increasing the reproductive success of the breeding pair (Van de Loock et al. 2017; Cousseau et al. 2022). As cooperative breeding affects maternal investment in placid greenbuls (Van de Loock et al. 2023), and thus may also affect immune function in both adults and nestlings (Valencia et al. 2006), we included cooperative breeding status as an explanatory variable in all statistical analyses. However, due to sample size limitations, we were unable to include an interaction between habitat quality and cooperative breeding, and therefore we were not able to formally test whether cooperative breeding may buffer the negative effects of habitat quality on immune function.

### Sampling

Nests and breeding groups were monitored during two breeding seasons in 2018/19 and 2021/22. Nests were checked every four to five days. When nestlings were five to seven days old, breeding females, breeding males and subordinates were captured with mist nets near the nest (*n* = 143 individuals at 79 nests). A blood sample (~ 60 µl) from a subset of these individuals (see below) was collected within 15 min of capture by venipuncture of the wing vein using heparinized capillaries. Individuals were fitted with numbered aluminium rings, a combination of three unique colour bands, weighed, measured (wing, tarsus, and head length) and checked for moult. The breeding status of each group member was determined by the presence of a brood patch (only dominant females incubate) or cloacal swelling (largest in dominant males). Immediately after release, the nest was recorded with a high-resolution video camera (Sony FDR-AX53) for three hours to determine the return latency of the released individuals and other flock members. To determine undisturbed feeding behaviour, nests were again recorded for five hours between 7 am and 2 pm when nestlings were approximately 9 days old (range 8–13 days). The camera was mounted on a tripod, positioned approximately 1,5 m from the nest, and protected and camouflaged by a waterproof cover. After video recording on nestling day 9, the nestlings were briefly removed from the nest and a small blood sample (< 40 µl, *n* = 52) was taken as described for adults. Nestlings were metal- and colour-banded, and their body mass, wing and tarsus lengths were measured (*n* = 115 nestlings at 69 nests). Cooperative breeding was determined by the presence of helpers observed feeding nestlings during videos taken on day 9. Feeding rates were calculated based on the total number of feeding events observed in five-hour videos and expressed as feeding rates per hour per nestling.

Blood samples were stored on ice until centrifuged for 10 min in a Spectrafuge Mini Laboratory Centrifuge (Labnet International, Inc.) on return from the field the same day. Plasma for BKA was stored at −20 °C until transport to the laboratory, where it was stored at −30 °C until analysis.

### Bacteria killing assay

To assess the strength of the placid greenbuls’ innate immune function, we measured the BKC of plasma against *Escherichia coli*. BKC is an integrative measure of baseline (constitutive) innate immune function (Millet et al. 2007; French and Neuman-Lee 2012), an animal’s first line of defence against foreign harmful bacteria, viruses, and other pathogens. The BKA procedure followed the protocol of French and Neuman-Lee (2012) with modifications by Eikenaar and Hegemann (2016), as well as adjustments of volume and bacterial concentrations to suit our study species. In accordance with Austrian safety regulations, *E. coli* strain #23,716 was used in this study, which does not require the use of a high safety level laboratory and has been successfully used in BKAs in previous studies (Millet et al. 2007).

Bacterial concentrations and plasma dilutions were determined in three test assays using pools of adult and nestling plasma samples. Final assays were performed using 4 µl plasma, 8 µl PBS and 4 µl bacterial solution with a concentration of 10^5^ *E. coli* cells per ml for adult birds. As innate immune function was found to be much weaker in nestlings, assays were performed using 6 µl of plasma and 6 µl of PBS to make 1:2 dilutions to which 4 µl of bacterial solution at a concentration of 10^4^ cells per ml was added for nestling samples. Samples were run in triplicate whenever possible, i.e., depending on the initial sample volume (92% of adult samples, 63% of nestling samples run in triplicate), remaining samples were run in duplicate. Two positive and two negative controls were added to the first and last column of each assay plate (total of eight controls per plate). Positive controls contained only tryptic soy broth, PBS, and bacterial solution, whereas negative controls contained only PBS and medium. Absorbance at 600 nm was measured once per hour for a total of 13 h using a Synergy HTX multi-mode reader (BioTek Instruments).

Adult and nestling samples were analysed separately. Samples from the 2018/19 breeding season were analysed in March 2021 (i.e., had been frozen for ~ 27 months) and samples from the 2021/22 breeding season were analysed in July 2022 (i.e., had been frozen for ~ 4 months). Although sample storage time can decrease immunological titres, in previous studies titres of the same individuals have been shown to be highly correlated before and after prolonged storage and biological patterns to remain stable (Kay et al. 2025; Hegemann et al. 2017). Furthermore, because samples within a year were all treated in the same way, we expected no systematic bias related to our study questions. Only comparisons between years may be affected by different storage times, yet long storage times usually result in stable BKCs (Liebl & Martin, 2009, Hegemann et al. 2017).

### Bacteria killing capacity and bacterial growth

BKC was calculated from the absorbance measured at 12 h using the formula provided by French and Neuman-Lee (2012). Absorbance at 1 min was subtracted from each measurement to control for background absorbance and BKC was calculated as one minus the mean absorbance for all replicates of a sample, divided by the mean absorbance for the positive controls where no plasma was added, and multiplied by 100 to obtain the percentage of bacteria killed relative to the positive control. Samples with a coefficient of variation (CV) greater than 20% within replicates were re-examined and unusual replicates (i.e., with unusual growth patterns, such as OD-measures dropping between readings and then spiking again) were excluded from the calculations (6 adult samples, 2 nestling samples). In total, 87 adult samples and 52 nestling samples were used for the final analyses. The mean (± SD) % CV of replicates was 6,62 ± 4,61% for adults and 6,68 ± 4,51% for nestlings. BKC values were calculated on the basis of two replicates only for six adult samples and 14 nestling samples, whereas values were based on single replicates for one adult and five nestling samples across both breeding seasons. The inter-assay CV of the positive controls was 2,95% for assays containing adult samples and 5,01% for nestling assays.

As the BKC is a relative measure based on a comparison of bacterial growth in a plasma sample and bacterial growth in a positive control, variation among positive controls has the potential to introduce variation in the final BKC measures that is not explained by individual differences in the ability to remove bacteria from the blood. This may be particularly relevant for nestling samples where low initial bacterial concentrations increase the risk of measurement error or in the case of variation in sample storage times. Therefore, in addition to the BKC, the bacterial carrying capacity (k), i.e., the maximum possible bacterial population size, was calculated using the R package growthcurver ver. 0.3.1 (Sprouffske and Wagner 2016). As the carrying capacity is calculated based on the hourly absorbance measurements for each sample, it is not dependent on positive controls, and it is therefore more resistant to variations in bacterial growth between controls and replicates. The carrying capacity is expected to be inversely related to the BKC, with high carrying capacities indicating low BKC. Indeed, the BKC and carrying capacity of adult and nestling samples were highly correlated (Pearson’s product-moment correlation: adults: r(85) = −0,99, *p* < 0,001; nestlings: r(50) = −0,78, *p* < 0,001). The results of these analyses were used to support the results obtained by BKC calculation. As previous eco-physiological studies have exclusively used BKC, we mainly report results based on BKC.

### Forest fragmentation and degradation

Forest degradation affects the stratification and structure of vegetation. We therefore quantified vertical vegetation structure and canopy cover at the territory level (see details below). In addition, fragment size was determined to capture forest fragmentation. Fragment size was based on native forest boundary maps of the Taita Hills derived from airborne remote sensing imagery (Pellikka et al. 2009, 2018; Kung’u et al. 2023).

### Vertical vegetation structure

Vertical vegetation structure around nest sites was assessed in four subplots of 15 m radius per territory, i.e., a central subplot based on the actual nest locations and three additional subplots 50 m away from the central subplot (50 m south, 50 m north-east, 50 m north-west) in April and May 2021 and 2022, i.e., shortly after the end of the breeding season. In each subplot, the presence of vegetation (0/1) was recorded at five points within a circle of 0,5 m radius in five height intervals (0–1 m, 1–5 m, 5–9 m, 9–15 m, > 15 m), resulting in a total of 20 records per sampling plot. We estimated vertical vegetation structure by calculating the Shannon-Wiener diversity index over the five vegetation height intervals, and by summing all occurrences of vegetation above 9 m for the 20 sampling points per plot (Bibby et al. 2012). As these were highly correlated (r(143) = 0,8, *p* < 0,001), we only retained the latter for further analysis as it showed higher variation.

### LiDAR based canopy cover

We used airborne Light Detection And Ranging (LiDAR) data to determine the percentage of indigenous forest canopy cover at each nest site. LiDAR data were collected from an airplane in January–February 2014 and February 2015 (breeding season), at an average altitude of approximately 1450 m above ground level using a Leica ALS60 sensor (Adhikari et al. 2017). We classified LiDAR points into ground and non-ground points and computed a digital terrain model (DTM) at 1 m resolution using LAStools software (rapidlasso GmbH). In addition, point heights were normalized using the DTM to derive heights from ground level. To calculate percent canopy cover, we extracted normalized point clouds for circular areas of 0,79 ha (50 m radius) around nest sites. A 3 m height limit was applied to separate ground/understory and canopy returns (Adhikari et al. 2017), and canopy cover was defined as the ratio of the first returns from the canopy to all first returns (Heiskanen et al. 2015). All nest sites were located within indigenous cloud forest and had dense vegetation cover below 3 m, except in cases of large canopy gaps or when nests were located near the forest edge. Canopy cover was calculated using the lidR package (Roussel et al. 2020) in the R environment (RCoreTeam 2023).

### Statistical analyses

#### General procedure

Four individuals were caught twice in the same breeding season. For these, only one sample was considered. 67 and 20 adult samples and 31 and 21 nestling samples were included for the 2018/19 and 2021/22 breeding seasons respectively. Samples from 2018/19 were collected from 40 different nests, whereas samples from 2021/22 were collected from 15 different nests. Sample sizes between years differed due to logistic reasons, not because of variation in reproduction.

All statistical analyses were performed with R version 4.1.2 (RCoreTeam 2023), including the packages lme4 ver. 1.1.27.1 (Bates and Maechler 2013), lmerTest ver. 3.1.3 (Kuznetsova et al. 2017), plyr ver. 1.8.6 (Wickham 2011), dplyr ver. 1.0.7, and ggplot2 ver. 3.3.5 (Wickham 2016). Nest-ID was included as a random effect in all models to account for measurements from multiple members of a breeding group or nestlings captured at the same nest. Model fit was confirmed by visual assessment of the fitted residuals and the Q-Q plot of each model and by using R package DHARMa (Hartig 2024).

Residual body mass was calculated as the residuals of a linear regression between tarsus length and body mass, including social status instead of sex, which was not known for most subordinates, as a factor (Guindre-Parker and Rubenstein 2018; Bolopo et al. 2019). For each individual, the mean of two separate measurements of the individual’s right tarsus was used to minimize measurement error. One individual escaped before being weighed, and the average of previous measurements from previous breeding seasons was used instead (weight range for 5 previous measurements for this individual 30,2–31,0 g).

To avoid over-fitting of statistical models, environmental variables such as temperature, humidity, and capture date were evaluated graphically and excluded from further analyses as they showed no visible correlation with BKC (temperature: *r* = 0,05, humidity: *r* = 0,009, capture date: *r* = 0,16). Moult was not included as a variable as it overlapped with social status (no moult in breeding females, moderate in breeding males, high levels in subordinates). Number of nestlings per clutch was initially included in the models but was excluded from further analyses to improve model fit, as there was no significant relationship between number of nestlings and adult BKC (F_1,45_ = 0,06, *p* = 0,81).

#### Is adult bacteria killing capacity and residual body condition related to forest fragmentation and degradation?

Linear mixed-effects models (LMMs, function lmer in package lme4) were used to test whether forest fragmentation and degradation and social status (breeding female, breeding male, helper) were related to the strength of adult BKC. We also tested whether the number of helpers had an effect on the BKC of breeding males and females, using LMMs with the number of helpers at the nest as a covariate. All models included residual body mass, nestling age and, in the latter model, also sex as covariates. Furthermore, all models also included study year (two levels: 2018/19 and 2021/22) to account for variation in sample storage times (Hegemann et al. 2017). Similar models were built for the analysis of variation in residual body mass.

#### Is nestling bacteria killing capacity and residual body condition related to forest fragmentation and degradation?

For nestlings, we tested whether forest fragmentation and degradation, number of helpers, and feeding rates were related to BKC. In all models, BKC was used as the dependent variable, with forest fragmentation and degradation, residual body mass, and study year as fixed effects. Number of helpers and feeding rates were not included in the same models due to differences in sample size. The same analysis was also run with the carrying capacity of *E. coli* grown in nestling blood plasma as the dependent variable. Similar models were also built to analyse the variation in residual body mass. However, the number of helpers per nest was removed from the analysis as it was not significant (F_1,48.93_ = 0,02, *p* = 0,89), and because DHARMa detected a deviation in the residuals when the number of helpers was included in the model.

#### Is innate immune function related to the return probability of the breeding pair?

To test whether the probability of breeding males and females returning to their nest within three hours after a disturbance, i.e., after capture and sampling, varied with BKC, two models were run. First, a generalized linear mixed-effects model (GLMM, function glmer in package lme4) with a binomial error distribution and a logit link function was used. Observed return (yes/no) was included as a binary response variable. Second, the dataset was reduced to those individuals that returned to the nest within the video time and a linear model was used to relate return latencies to BKC. BKC as well as residual body mass, handling time (from capture to release), sex and study year (two levels 2018/19 and 2021/22) were included as covariates in all models.

## Results

### Is adult bacteria killing capacity and residual body mass related to forest fragmentation and degradation?

**Fig. 1:**
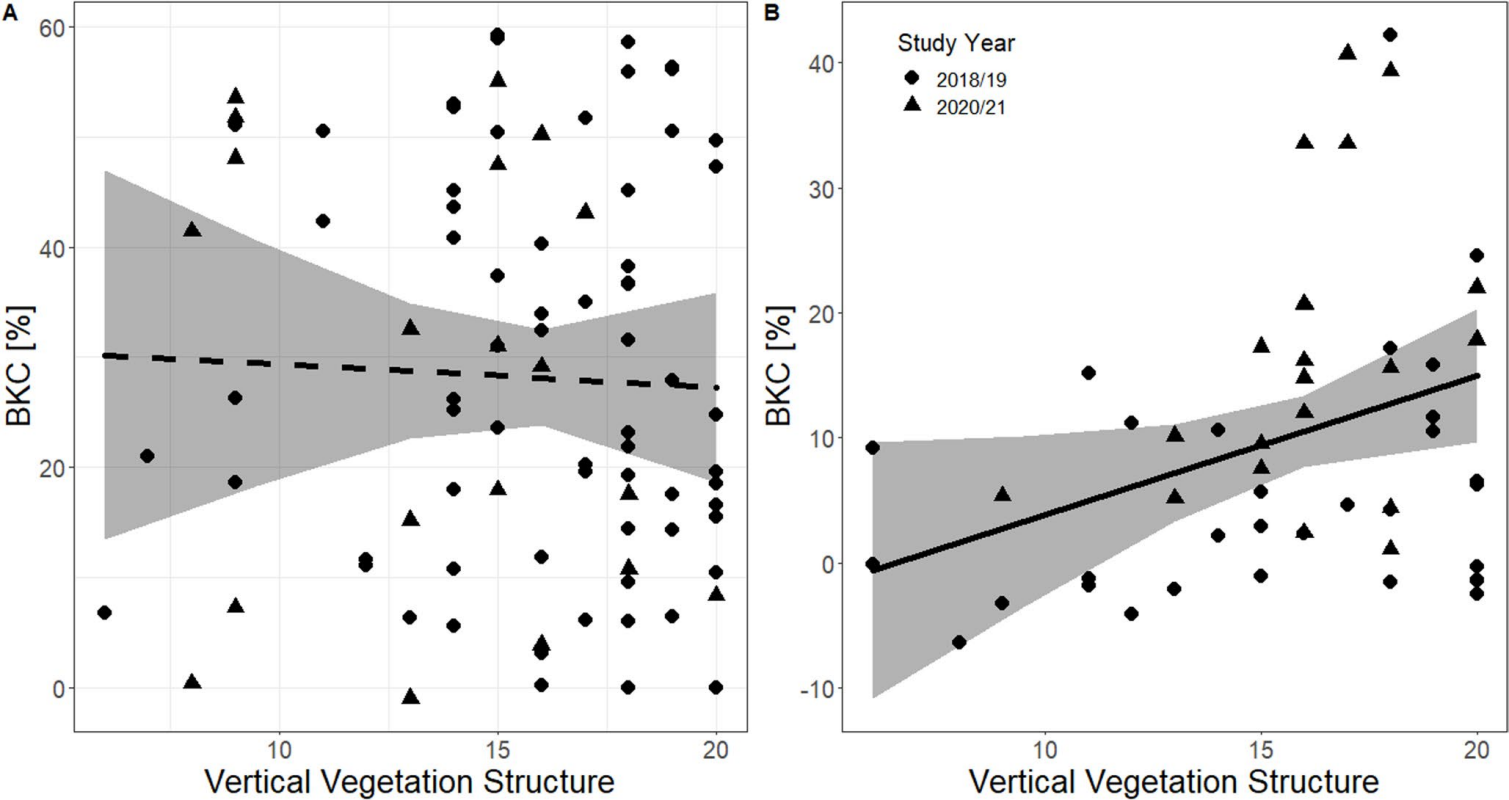
Effect of vertical vegetation structure on innate immune function as measured by bacterial killing capacity (BKC) of adult (**A**) and nestling (**B**) placid greenbuls. Shown are raw data points (dots), REML model predictions (line) and 95% confidence intervals (grey shading). Dashed line indicates a non-significant relationship. Shape indicates study years in which individuals were sampled

BKC in adults was not related to fragment size, canopy cover, or vertical vegetation structure (Table 1; Fig. 1A). Furthermore, the BKC of breeding males and females was not related to the number of helpers (F_1,42_ = 0,003, *p* = 0,96). Study year and thus sample storage time had no significant influence on adult BKC (Table 1). Residual body mass was not related to fragment size, canopy cover, or vertical vegetation structure (Table 1). Adults had higher residual body mass in 2020/21 than in 2018/19 and parents of older nestlings had lower residual body mass than those of younger nestlings (Table 1).

**Table 1:** Statistics and coefficients for linear mixed-effects models determining the relationship between variation in bacteria killing capacity (BKC) of blood plasma and residual body mass collected from adult placid greenbuls nesting in territories of varying quality in cloud forest fragments of the Kenyan Taita Hills and different measures of territory quality. CI, 95% confidence interval; DF, degrees of freedom. Significant relationships are presented in bold

	Estimate [CI]	F_DF_	*P*	Estimate [CI]	F_DF_	*P*
	BKC (87 observations at 51 nests)			Residual body mass (143 observations at 79 nests)		
(Intercept)	25,58 [9,17–41,99]		0,003	1,45 [0,47–2,42]		0,004
Residual Body Mass	2,70 [−0,07–5,48]	F = _1,66_ = 3,76	0,06			
Study Year	−1,09 [−13,40–11,22]	F_1,50_ = 0,03	0,86	**0**,**79** **[0**,**23–1**,**34]**	**F**_**1,135**_ **= 7**,**89**	**0**,**006**
Age Nestlings	0,27 [−1,75–2,30]	F_1,78_ = 0,07	0,79	**−0**,**24** **[−0**,**36 - −0**,**12]**	**F**_**1,135**_ **= 16**,**81**	**< 0**,**001**
Social Status - helper	7,29 [−3,22–17,79]	F_2,63_ = 2,00	0,14	0,56 [−0,08–1,20]	F_2,135_ = 1,50	0,23
Social Status - male	−2,83 [−11,67–6,01]			0,22 [−0,36–0,80]		
Canopy Cover (%)	−3,70 [−7,81–0,42]	F_1,65_ = 3,20	0,08	−0,08 [−0,36–0,19]	F_1,135_ = 0,35	0,55
Vertical Vegetation Structure	−0,73 [−6,67–5,21]	F_1,46_ = 0,06	0,81	0,10 [−0,19–0,39]	F_1,135_ = 0,44	0,51
Fragment Size	2,09 [−3,53–7,71]	F_1,48_ = 0,55	0,46	0,22 [−0,07–0,52]	F_1,135_ = 2,27	0,13

### Is nestling bacteria killing capacity and residual body mass related to forest fragmentation and degradation?

BKC in nestlings was positively correlated with vertical vegetation structure (Table 2; Fig. 1B), but there was no correlation between nestling BKC and fragment size or canopy cover. The number of helpers was not related to nestling BKC. Nestling BKC differed significantly between study years, with samples from 2021/22 breeding season having a stronger BKC than those from 2018/19. The carrying capacity of *E. coli* grown in nestling blood plasma was negatively correlated with vertical vegetation structure (Online Resource 1), which was expected as BKC and carrying capacity are inversely related. Nestling BKC was not significantly related to feeding rates (F_1,42_ = 0,38, *p* = 0,54). No significant relationship was found between residual body mass and fragment size or canopy cover (Table 2). Similarly, there was no significant relationship with vertical vegetation structure, though a non-significant trend was observed in the same direction as bacteria killing capacity (Table 2). Nestling residual body mass did not vary between study years (Table 2).

**Table 2:** Statistics and coefficients for linear mixed-effects models determining the relationship between variation in bacteria killing capacity (BKC) of blood plasma collected from nestling placid greenbuls hatched in differently sized groups in cloud forest fragments of the Kenyan Taita Hills and different measures of territory quality. CI, 95% confidence interval; DF, degrees of freedom. P-values < 0,05 are in bold

	Estimate [CI]	F_DF_	*P*	Estimate [CI]	F_DF_	*P*
	BKC (52 observations at 36 nests)			Residual body mass (115 observations at 69 nests)		
(Intercept)	−0,27 [−11,67–11,13]		0,96	0,26 [−0,43–0,95]		0,46
Residual Body Mass	1,86 [−0,41–4,13]	F_1,45_ = 2,74	0,10			
Number of Helpers	1,90 [−1,22–5,02]	F_1,45_ = 1,51	0,22	−0,03 [−0,38–0,32]	F_1,49_ = 0,03	0,87
Study Year	**10**,**66** **[4**,**12–17**,**19]**	**F**_**1,45**_ **= 10**,**81**	**0**,**002**	0,35 [−0,37–1,08]	F_1,59_ = 0,94	0,34
Canopy Cover (%)	−0,03 [−2,58–2,52]	F_1,45_ = 0,00	0,98	0,06 [−0,31–0,43]	F_1,56_ = 0,09	0,77
Vertical Vegetation Structure	**4**,**13 [0**,**41–7**,**85]**	**F**_**1,45**_ **= 5**,**00**	**0**,**03**	0,33 [−0,08–0,74]	F_1,60_ = 2,49	0,12
Fragment Size	−3,33 [−7,40–0,74]	F_1,45_ = 2,73	0,11	−0,14 [−0,55–0,28]	F_1,61_ = 0,42	0,52

### Is innate immune function related to the return probability of the breeding pair?

**Fig. 2:**
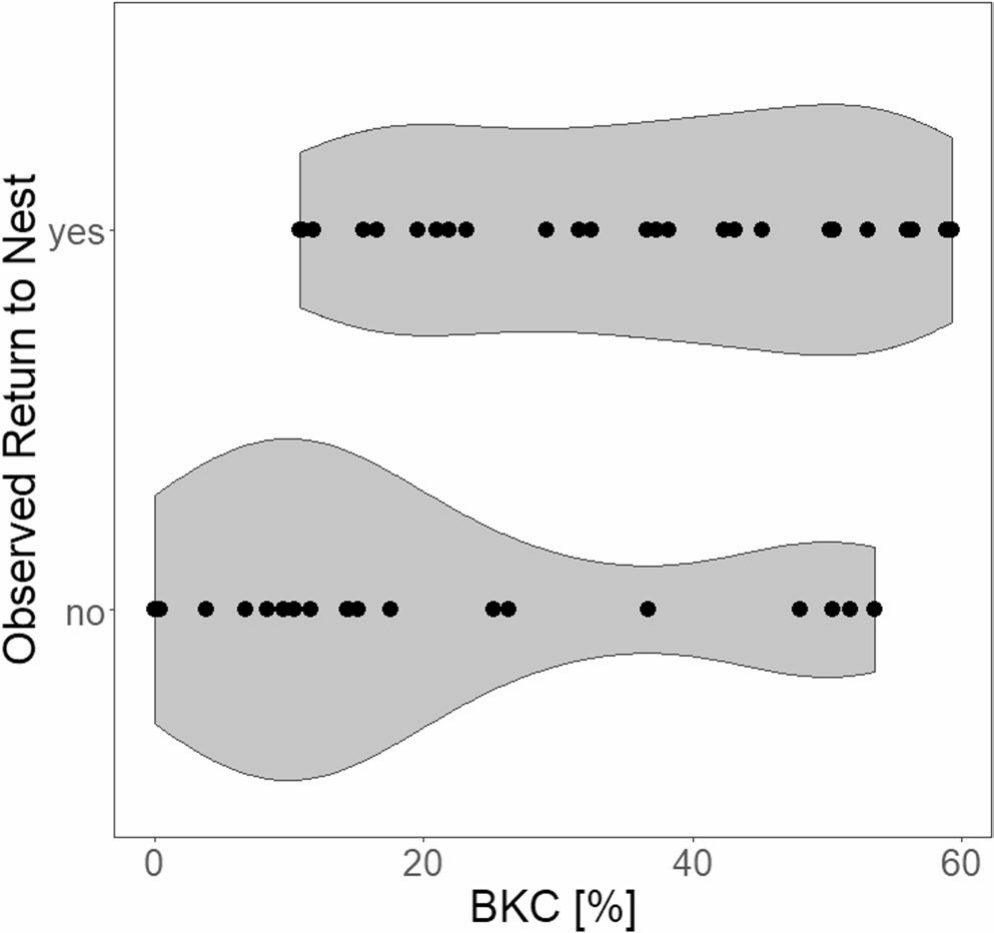
Frequency of observed return to nest after disturbance in relation to individual BKC of breeding males and females during the 2018/19 and 2021/22 breeding seasons. Plot area indicates frequency at which response was observed at corresponding bacterial killing capacity (BKC). Dots show raw data points

Breeding males and females that returned to the nest within three hours of capture and handling had significantly higher BKC than breeders that did not return within three hours (Table 3; Fig. 2). Longer handling times also reduced the likelihood of individuals returning to the nest after release (Table 3) and individuals sampled in study year 2021/22 on average took longer to return to the nest (Table 3). Amongst those individuals that returned to the nest within the video time, those with shorter return latencies had significantly higher BKC than those with longer return latencies (*n* = 29, −1,30 [−2,33 - −0,27], t = −2,62, *p* = 0,02).

**Table 3:** Summary of statistics and coefficients for binomial generalized linear mixed-effects model on the probability of breeding males and females returning to the nest after disturbance (capture and sampling). P-values < 0,05 are in bold

	Estimate	SE	z	*p*
(Intercept)	4,10	2,27	1,80	0,07
BKC_12h	**0,06**	**0,02**	**2,53**	**0,01**
Handling Time	**−0,26**	**0,12**	**−2,07**	**0,04**
Sex*	−0,43	0,90	−0,48	0,63
Residual Body Mass	0,21	0,34	0,64	0,52
Study Year**	**−2,13**	**0,99**	**−2,15**	**0**,**03**

* Sex: female is reference

** Study Year: 2018/19 is reference

## Discussion

BKC, an integrative measure of baseline (constitutive) innate immune function, was related to forest degradation in placid greenbul nestlings but not in adults. Specifically, nestling immune function was lower in territories with less well-developed vertical vegetation structure, a proxy for forest integrity. In addition, breeding males and females with a lower BKC were less likely to return to their nest immediately after disturbance, consistent with the prediction that individuals in poor condition (i.e., low BKC) are more sensitive to stressors.

Habitat change may affect diverse aspects of an animal’s life. In previous studies we have shown that placid greenbuls travel longer distances between foraging patches, have larger home ranges, and higher corticosterone levels in more degraded forest sites (Apfelbeck et al. 2024; Kung’u et al. 2025). In contrast, in the present study, we did not find that habitat degradation was related to an integrative measure of baseline innate immune function or residual body mass in breeding placid greenbuls. This suggests that although adult placid greenbuls show behavioural and physiological changes during breeding that indicate increased reproductive workload and energetic demands, their body condition, here measured as immune function and residual body mass, was not compromised. Instead, BKC of nestlings was positively correlated with vertical vegetation structure, i.e., nestlings born in low quality forest sites had a lower index of immune function than those born in high quality ones. In addition, although the present study only showed a non-significant positive trend between residual body mass and vertical vegetation cover, an analysis of long-term morphological data on body condition in nestling greenbuls revealed a positive association between habitat quality and nestling body condition (Kung’u et al. under review). Thus, our results indicate that nestlings could be the ones paying the (immune) cost of breeding in degraded forest sites, which may further translate to reduced survival costs (Eraud et al. 2009, Pitala et al. 2010, Hegemann et al. 2013) and have consequences for species persistence unless supplemented by immigrating adults from higher quality source habitats.

Forest fragmentation and degradation have been shown to alter important resources, such as insect abundance (Zanette et al. 2000; Kung’u et al. 2023) and arthropod communities (Morante-Filho et al. 2016). When facing reduced food resources, breeders may pass on the burden of increased foraging costs in degraded habitat (increased distances between foraging patches, larger home ranges, increased corticosterone levels) to their offspring by reducing nestling provisioning rates. However, there is no evidence of such an adjustment in placid greenbuls (i.e., parental provisioning rates are not related to territory quality, Kung’u et al. under review) and there was no evidence for a relation between feeding rates and BKC as a measure of nestling innate immunity in the present study. In addition, the number of helpers was not associated with nestling immunity, although their presence increases overall feeding rates in placid greenbuls (Van de Loock et al. 2023). However, whereas parents and helpers may be able to keep feeding frequency constant when faced with reduced resources, food quality may be reduced (Wright and Cotton 1998). Neither the quantity nor the quality of the food provided to nestlings was assessed, though this may influence nestling immune function as shown in several other species (Lochmiller et al. 1993; Klasing 1998; Birkhead et al. 1999; Hegemann et al. 2013). Alternatively, as nest predation is a common cause of nest failure in this species (Spanhove et al. 2014), any additional food provided by helpers may be invested in physical growth to fledge early from the nest, rather than in increased development of immune function. Consistent with this, nestling wing length, an important predictor of post-fledging survival in many species (Jones and Ward 2020; Aastrup et al. 2023) has been shown to be positively correlated with the number of helpers at the nest (Van de Loock et al. 2023).

Placid greenbuls and other cooperatively breeding species reduce feeding rates in the presence of helpers (Downing et al. 2021; Van de Loock et al. 2023), and the presence of helpers has a positive effect on annual survival and lifespan of the breeding pair (Hammers et al. 2019; Downing et al. 2021; Cousseau et al. 2022). Although studies have found a positive effect of helpers on oxidative status (Cram et al. 2015) and glucocorticoids (Apfelbeck et al. 2024), we did not find a relationship between immune function, estimated using BKC, and cooperation in breeding placid greenbuls. Positive effects of cooperation on individual condition may only appear when individuals are facing challenging conditions, such as when habitat quality is low (Apfelbeck et al. 2024) or when reproductive effort is experimentally increased (Cram et al. 2015). However, due to a limited sample size, we were unable to consider the interaction between habitat quality and cooperative behaviour on immune function in the present study. Alternatively, these results may indicate that breeders invest in a strong immune function regardless of breeding conditions, i.e., territory quality or the presence of helpers.

Furthermore, our findings suggest that immune function strength influences reproductive decision making in placid greenbuls as breeders with a weaker immune function as measured by BKC were significantly less likely to return to the nest after capture. This is consistent with the hypothesis that breeders in long-lived species are more likely to invest in their own well-being and thus potentially compromise nestling development in a trade-off between self-maintenance and current reproductive output. Immunological constraints may therefore be an important factor in regulating parental investment and individuals with a weaker immune function may prioritise their own survival over offspring care. This is consistent with findings in other long-lived species, such as seabirds, where parents have been shown to regulate their reproductive effort or abandon broods in favour of self-maintenance (Sæther et al. 1993; Weimerskirch et al. 1995, Erikstad et al. 1998). In contrast, in short-lived species, low quality parents have been shown to reduce their own immunocompetence in favour of offspring quality (Ardia 2005). In addition to individual condition, environmental variability can also influence whether individuals prioritize self over current reproduction (Breuner and Hahn 2003). We have previously shown that placid greenbuls in the smallest fragments are less likely to return to the nest after capture than those from the larger fragments (Apfelbeck et al. 2024). Thus, both individual condition and habitat quality influence reproductive decision making in placid greenbuls as in other species.

A meta-analysis found that many mammals and birds show strong immunological responses to habitat degradation (Messina et al. 2018). Interestingly, all of the bird studies included in this meta-analysis were conducted in the northern hemisphere, whereas no studies of how habitat degradation might impact immune function in tropical bird species have been available to date. As temperate and tropical bird species often differ in their pace of life, i.e., tropical species generally have lower reproductive investments (Cardillo 2002) but higher annual survival probabilities (Muñoz et al. 2018), they can be expected to resolve the trade-off between current reproduction and self-maintenance differently. As the placid greenbul is a comparatively long-lived species (Cousseau et al. 2020), individuals are expected to prioritise self-maintenance and future reproductive output over current reproductive effort when conditions for breeding are challenging (Tieleman et al. 2008).

While storage times differed for samples from different years, no difference in BKC was found for adults. In contrast, nestling BKC varied between years with higher BKC in those samples that were stored for a shorter time. However, nestling carrying capacity was similar between study years suggesting that this is a useful approach to control for such variation. Furthermore, although differences in storage times may have affected the overall BKC level in nestlings, biological patterns remained (Hegemann et al. 2017). In addition, as adult samples did not show such variation, it is also conceivable that other, i.e., environmental factors, might have affected BKC in nestlings, especially as we also found other variables, such as residual body mass, to differ between study years.

## Conclusion

This study demonstrated that territory quality was not related to innate immune function in adult placid greenbuls, whereas nestlings instead appeared to bear the costs of breeding in disturbed habitats. This pattern was somewhat unexpected because our previous studies showed clear behavioural and physiological consequences of breeding in poor habitat for adult placid greenbuls, including increased home ranges and elevated corticosterone levels (Apfelbeck et al. 2024; Kung’u et al. 2025). These present results suggest that adults even in degraded forests minimized disease-related mortality risk by maintaining strong immune defences. The causal link between habitat degradation and reduced nestling condition (immune function (this study), scaled mass index (Kung’u et al. under review)) remains to be determined as variation in provisioning rates was not related to territory quality in placid greenbuls (Kung’u et al. under review). Furthermore, because of the correlative nature of our study and the complexity of immune function, experimental studies will need to unravel causal relationships. Yet, our findings highlight the importance of studying the effects of habitat fragmentation and degradation in the tropics, where most of the global biodiversity occurs, as tropical species may respond differently to temperate species due to life history differences.

## Supplementary Information

Below is the link to the electronic supplementary material.

Supplementary Material 1 (DOCX 47.5 KB)

## Data Availability

The data that support the findings of this study are available in Mendeley Data with the identifier https://doi.org/10.17632/zn7w69kjsc.1
