# Supplementary material for: Habitat degradation relates to reduced immune function in nestlings, but not adults, of a tropical forest bird
Source: Naturwissenschaften. 2025 Nov 26;112(6):90. doi: 10.1007/s00114-025-02046-3 (PMC12657580; doi:10.1007/s00114-025-02046-3)
Supplement: Supplementary file 1 — Supplementary Material 1 (DOCX 47.5 KB) [file 114_2025_2046_MOESM1_ESM.docx]

Habitat degradation relates to reduced immune function in nestlings, but not adults, of a tropical forest bird

The Science of Nature

Tamara Isabelle Sorg ^1,2,3^, Arne Hegemann ^4,12^, Laurence Cousseau^5,8^, Gladys Nyakeru Kung’u^1,8^, Janne Heiskanen^6,9^, Petri Pellikka^6,10,11^, Mwangi Githiru^7,8^, Luc Lens^5^, Beate Apfelbeck^1,8*^^[[1]](#footnote-1)^#

^1^Department of Environment and Biodiversity, University of Salzburg, 5020 Salzburg, Austria

^2^Behavioral Ecology and Sociobiology Unit, German Primate Center, 37077 Göttingen, Germany

^3^Department of Sociobiology/Anthropology, Johann-Friedrich-Blumenbach Institute of Zoology and Anthropology, Georg-August-University Göttingen, 37077 Göttingen, Germany

^4^Department of Biology, Lund University, 223 62 Lund, Sweden

^5^Centre for Research on Ecology, Cognition and Behaviour of Birds, Ghent University, 9000 Ghent, Belgium

^6^Department of Geosciences and Geography, University of Helsinki, 00014, Finland

^7^Wildlife Works, P.O. Box 310-80300, Voi, Kenya

^8^Zoology Department, National Museums of Kenya, P.O. Box 40658 – 00100, Nairobi, Kenya

^9^Finnish Meteorological Institute, P.O. Box 503, 00101 Helsinki, Finland

^10^Finnish Southern Africa Cooperation Institute, 10 Schwabe Street, Windhoek, Namibia

^11^State Key Laboratory for Information Engineering in Surveying, Mapping and Remote Sensing, Wuhan University, Wuhan 430079, China

^12^currrent address: Institute of International Animal Health / One Health, Friedrich-Loeffler-Institute, Federal Research Institute for Animal Health, Südufer 10, 17493 Greifswald - Insel Riems, Germany

*Corresponding author: [beateanna.apfelbeck@plus.ac.at](mailto:beateanna.apfelbeck@plus.ac.at), +4366280445512

**Appendix 1** Summary of statistics and coefficients for linear mixed-effects models determining relationships between variation in carrying capacities of E. coli grown in blood plasma as a measure of the strength of the innate immune function and different measures of territory quality of nestling placid greenbuls hatched in differently sized groups in cloud-forest fragments of the Kenyan Taita Hills. P-values < 0,05 are in bold.

|  | Estimate | CI | DenDF | F | P |
| --- | --- | --- | --- | --- | --- |
| (Intercept) | 1,19 | 1,06 – 1,31 | 45 |  | <0,001 |
| Residual Body Mass | -0,01 | -0,04 – 0,01 | 45 | 0,99 | 0,28 |
| Number of Helpers | -0,05 | -0,08 – -0,01 | 45 | 6,84 | **0,01** |
| Study Year | -0,03 | -0,11 – 0,04 | 45 | 0,87 | 0,36 |
| Canopy Cover (%) | 0,00 | -0,03 – 0,03 | 45 | 0,02 | 0,88 |
| Vertical Vegetation Structure | -0,06 | -0,10 – -0,01 | 45 | 7,34 | **0,01** |
| Fragment Size | 0,04 | -0,00 – 0,09 | 45 | 3,37 | 0,07 |

## **Appendix 2** Reliability of thawed samples

Although a previous study by (Jacobs and Fair 2016) urged to perform bacteria killing assays using only fresh blood samples instead of frozen ones, a contrasting study by (Hegemann et al. 2017) found no strong detrimental effect of repeated thawing and refreezing on the bacteria killing ability of blood samples. After some of our samples, which were not used in the final analyses of this study, were unintentionally thawed during transport and could not be immediately refrozen, they showed a clear difference to samples that had been continuously frozen throughout transport. The thawed samples in our study were considered unreliable and excluded from further analysis and therefore not included, highlighting the importance of ensuring stable conditions during transport and handling when working with frozen blood samples.

Hegemann, A., S. Pardal, and K. D. Matson. 2017. Indices of immune function used by ecologists are mostly unaffected by repeated freeze-thaw cycles and methodological deviations. Front Zool **14**:43.

Jacobs, A. C., and J. M. Fair. 2016. Bacteria-killing ability of fresh blood plasma compared to frozen blood plasma. Comp Biochem Physiol A Mol Integr Physiol **191**:115-118.

1. [↑](#footnote-ref-1)
